# Exploring the potential of cognitive intra-individual variability in HIV care and research in adults with HIV: a concept analysis

**DOI:** 10.1080/23279095.2026.2694678

**Published:** 2026-07-03

**Authors:** Hathaichanok Phaowiriya, Victor A. Del Bene, Kun Wang, Pamela G. Bowen, Elizabeth Byrd, Susan Egas, Maryam Rostamvand, Heather Shelton, Xueling Zeng, Pariya L. Fazeli, David E. Vance

**Affiliations:** a School of Nursing, University of Alabama at Birmingham, Birmingham, Alabama, USA; b Princess Agrarajakumari Faculty of Nursing, Chulabhorn Royal Academy, Bangkok, Thailand; c Heersink School of Medicine, University of Alabama at Birmingham, Birmingham, Alabama, USA; d Department of Social Work, University of Alabama at Birmingham, Birmingham, Alabama, USA; e Department of Psychology, University of Alabama at Birmingham, Birmingham, Alabama, USA

**Keywords:** Cognition, cognitive IIV, dispersion, HIV care, intra-individual variability

## Abstract

Cognitive intraindividual variability (IIV) refers to the variability in one’s ability to sustain consistent cognitive performance. This phenomenon is linked to the emergence of cognitive impairment and dementia. The aim of this concept analysis using Rodger’s evolutionary approach was to clarify the unique characteristics of the concept of cognitive IIV in HIV care and research. PubMed, Embase, CINAHL, and PsycINFO were utilized which yielded 22 articles that were analyzed to inform the concept analysis. After considering antecedents, attributes, and consequences, many antecedents may also function as consequences, depending on how they influence or are influenced within the cause-and-effect process. This concept analysis highlights the potential of cognitive IIV as a sensitive indicator of cognitive health in people with HIV (PWH). A better understanding of the variation of cognitive performance in PWH will assist mental health professionals in developing more tailored interventions and achieving improved clinical outcomes.

Fifty percent of people with HIV (PWH) in the United States are currently 50 and older, and by the year 2030, 70% are expected to be 50 and older (Wing, 2017). With the rise in the number of older PWH, concerns grow that age-related cognitive impairments could exacerbate disease-related cognitive impairments, further affecting daily functioning (Vance, Lang, et al., 2025). HIV-associated neurocognitive disorders (HAND) affect approximately 20–60% of PWH, depending on diagnostic criteria; however, these criteria can also classify over 20% of people without HIV (PWoH) as impaired, which reflects the limitations in measurement rather than disease-specific effects. Therefore, while PWH have a higher prevalence of HAND, the difference reflects an elevated risk rather than a distinct or exclusive condition (Nightingale et al., 2023). In fact, in a recent study in a larger cohort sample of men and women with and without HIV (N = 2,684) found that in PWH and PWoH, cognitive performance was largely comparable and serostatus differences were relatively small (effect sizes < 0.15) (Rubin et al., 2026). Moreover, aging with HIV presents additional challenges to brain health from age-related neurodegeneration, chronic inflammation, and comorbidities, even when effectively managed with antiretroviral therapy (ART) (Chan & Valcour, 2022).

Cognition is typically evaluated in clinical and research settings using standardized cognitive assessments across important domains of cognition (e.g., attention, language). These assessments measure one’s average level of performance, providing an overview of one’s cognitive ability (Del Bene et al., 2023). For example, a participant completes a memory recall task or problem-solving activity, and their mean score or total score (depending on the test) is used to determine whether their cognitive function is in the normal, impaired, or at-risk range. While it is useful to quantify levels of function using cognitive tests, traditional mean-based cognitive assessments do not capture variability in cognitive performance that can signal early executive dysfunction or subtle cognitive impairment (Penheiro, Webber, et al., 2025; Ridgely et al., 2024; Vance et al., 2022). In this context, cognitive intra-individual variability (IIV) becomes conceptually, methodologically, and clinically relevant.

Cognitive IIV refers to the variability in cognitive performance across different cognitive domains (dispersion) or even within a single cognitive test over a brief interval (inconsistency), and is associated with the onset of cognitive impairment in various clinical populations (e.g., dementia, schizophrenia, white matter disease, bipolar disorder, ADHD) (Caruana et al., 2025; Cole et al., 2011; Frazier-Wood et al., 2012; Jones et al., 2018; Vance et al., 2022). Based on prior research, greater cognitive IIV can be an early warning sign of the brain’s inability to plan, focus, and regulate behavior, even if their mean-based cognitive scores are normal (Del Bene, Aita, et al., 2025; Kiselica, Karr, et al., 2024). For an example of dispersion, as presented in Figure 1A, Maryam and Heather completed a cognitive battery that examined seven cognitive domains (i.e., executive function, attention, speed of processing, verbal learning, verbal memory, visual learning, and visual memory) and had the same composite mean score (e.g., T-score = 45). This could suggest that they are performing at a comparable level. Albeit, when looking at the differences in scores across domains, Maryam showed little variability in cognitive performance across these cognitive domains, while Heather expressed more variability across cognitive domains. Based on the concept of cognitive IIV, this spread of cognitive performance (i.e., dispersion) could suggest that Heather has a higher risk for cognitive decline than Maryam. In this example of dispersion, we recognize that the use of a composite score does not reflect how clinicians typically interpret neuropsychological data in practice; however, calculating the composite score is considered an essential first step for clinicians who want to use cognitive IIV, and it is impossible to calculate cognitive IIV without the composite score. Additionally, the differences in scores across cognitive domains, which are represented as the multiple points of cognition, were used to determine the variability across cognitive domains. If there is more variability between cognitive domains, the cognitive IIV score increases. Moreover, some may prefer to remove the outliers to have a more stable measure of cognitive IIV, which may or may not contribute to bias in how it is measured. In applied clinical work, a comprehensive neuropsychological assessment provides detailed, domain-specific information rather than relying on a single aggregate measure of overall performance. Accordingly, dispersion is presented here as a summary indicator of variability, but it should be understood as complementary to, rather than a substitute for, the detailed, domain-level neuropsychological evaluation. A further underlying theoretical consideration is that having a deficit in a cognitive domain does not by itself constitute cognitive IIV, rather it is the spread of both high and low cognitive domain scores, the overall fluctuation, in which cognitive IIV is measured. Currently, cognitive IIV is primarily used in the research setting rather than a clinical setting. Yet, regarding its real word application, greater dispersion has been shown to predict adverse clinical outcomes in PWH, including cognitive decline, functional impairment, poorer adherence to ART adherence, and increased mortality (Anderson et al., 2018; Arce Renteria et al., 2020; Del Bene, Fazeli, et al., 2025).

Similarly, Figure 1B illustrates inconsistency, which is related but distinct from dispersion, reflects variability within a single cognitive domain. In a reaction time test (i.e., a measure of attention) with multiple trials, Maryam and Heather have the same mean reaction time (e.g., 0.67 seconds) but differ in the consistency of their responses. Maryam shows a more consistent reaction time, while Heather shows more variability. Here, the same interpretation as dispersion applies: the greater the inconsistency, the poorer the predicted cognitive outcomes over time (Del Bene, Aita, et al., 2025). Therefore, measuring cognitive IIV provides a complementary measure of cognitive performance that traditional mean-based assessments miss. In other words, a good way to think about cognitive IIV is that this is the standard deviation of a mean. In research, we need to know about the distribution around the mean to make a meaningful interpretation of the data. The same goes for interpreting this information clinically; both are important and provide useful information to contextualize the findings. Further, greater inconsistency has been shown in many HIV studies to predict cognitive decline, functional impairment, and reduced ART adherence (Ettenhofer et al., 2010; Harrison et al., 2017; Odii et al., 2025; Penheiro, Webber, et al., 2025).

Most studies operationalized cognitive IIV by using two different mathematical formulae to calculate it, including: (1) iSD, and (2) CoV. The iSD reflects the raw standard deviation of an individual’s performance across multiple cognitive tasks, capturing absolute variability without adjusting for overall mean performance. On the other hand, the CoV represents the ratio of the iSD divided by the mean scores of performances across multiple cognitive tasks, thereby accounting for individual differences in average performance levels. While both methods reflect cognitive IIV, they have slightly different predictive values (Vance et al., in press).

The Executive Dyscontrol Hypothesis posits that cognitive IIV arises when there are disruptions in executive control processes, which are primarily controlled by the prefrontal cortex (Martínez et al., 2016). Other brain areas have also been implicated in cognitive IIV, including the cerebellum, cingulate cortex, and basal ganglia (Wang et al., 2019; Zhang et al., 2023; Zuo et al., 2023). This hypothesis is particularly relevant for PWH, who faced increased risk of cognitive impairments due to the effects of the virus, neurotoxicity from HIV medications, associated psychosocial stressors, and aging-related comorbidities (Vance et al., 2022). These factors (or disruptions) affect the brain’s ability to consistently regulate cognitive processes, resulting in increased moment-to-moment variability in cognitive performance. This has real world consequences. For instance, in a study of 46 PWH in a driving simulator, researchers found that older PWH (*M* = 51.23, *SD* = 6.17) who experience poorer speed of processing, which is vulnerable to cognitive IIV, were more prone to unsafe driving behaviors and accidents (Vance et al., 2014).

Despite its relevance in other clinical populations, cognitive IIV is a relatively new concept in HIV care and research. Applying the Executive Dyscontrol Hypothesis (Penheiro, Webber, et al., 2025) offers a valuable neurocognitive framework for exploring why PWH may experience either moment-to-moment variability in cognitive abilities or deficits in some cognitive domains but not others. This approach can assist in identifying the psychosocial and clinical factors that impact executive functioning, more specifically cognitive IIV, and connecting cognitive IIV to important real-life outcomes, such as medication adherence and the ability to live independently. The Executive Dyscontrol Hypothesis not only explains the underlying mechanisms of cognitive IIV but also suggests that greater variability may serve as an early marker of cognitive decline (Vance et al., 2022). Previous studies have found that greater cognitive IIV in PWH is associated with worse cognitive performance and decline over time, as well as poorer everyday functioning, more comorbidities, and higher mortality risk (Anderson et al., 2018; Vance et al., 2022).

The purpose of this article is to clarify the meaning, attributes, antecedents, and consequences of cognitive IIV and to evaluate its potential role in HIV care and research. Using Rodgers’ evolutionary concept analysis method (Rodgers & Knafl, 2000), we defined the cognitive IIV within the context of HIV and support its integration into future clinical and research frameworks targeting cognitive health in PWH. To achieve this, we: (1) identified surrogate terms, related concepts, antecedents, attributes, and consequences of cognitive IIV in HIV care and research; (2) provided a model to illustrate cognitive IIV; (3) constructed a theoretical definition; and (4) discussed implications for clinical practice and research.

## Methods

### Overview

Rodgers’ evolutionary method was selected to guide this concept analysis because it recognizes that concepts are dynamic and ever changing, thus needing to be refined and expanded (Foley & Davis, 2017). This approach involved systematically identifying and synthesizing key attributes, antecedents, and consequences from the literature, an approach not a part of typical narrative or systematic reviews. The relevant studies were reviewed and coded iteratively. Then, the recurring concepts were grouped into themes relevant to antecedents, attributes, and consequences related to the concept by the investigators who discuss and consolidate the concepts. This method was selected because it allows for the integration of diverse conceptualizations and methodologies in the HIV and cognitive IIV literature. Albeit, this approach may involve some subjectivity in the theme identification and is dependent on the scope and quality of the available literature.

Cognitive IIV research is growing in popularity as a relatively new strategy to understand the nuances of patient’s cognitive data; furthermore, how it is measured is continually being refined within a research and clinical setting (Del Bene, Aita, et al., 2025). For dispersion in particular, no standard exists for how many cognitive measures are needed to calculate it. If only a few measures are used, the estimate may be unstable and fail to adequately capture variability across domains. Many dispersion studies typically use approximately five to six cognitive measures to capture cognitive IIV across cognitive domains (e.g., Klepacz et al., 2025). Additionally, dispersion is operationalized as variability across a battery of neuropsychological tests that often comprise multiple measures across several cognitive domains (Kiselica et al., 2024). Thus, based on a current state of the science article on cognitive IIV, Del Bene, Aita, et al. (2025) posit that the number and type of cognitive domains included in assessment vary by clinical population and may influence cognitive IIV estimates, while small sets of measures (e.g., 4–6 tests) can yield comparable results, and using more tests across multiple domains is recommended to improve reliability and reduce measurement error. In a meta-analysis on cognitive IIV in Alzheimer’s disease, the number of tests used in the IIV calculation did not bias the group differences (Aita et al., 2025). Albeit, cognitive IIV has yet to be integrated into clinical research or practice. It is potentiality as adjunctive cognitive information is relatively new and not well understood. Furthermore, most mental health professionals are not expected to carry out full neuropsychological assessments because it requires specialized training, licensure, and postdoctoral training in neuropsychology. Full assessments of cognitive IIV require the comprehensive processes involving administration, scoring, and interpretation, making them impractical for routine clinical practice.

Additionally, one of the key methodological difficulties is that differences in how cognitive IIV is defined and calculated across studies poses challenges for synthesis and comparison. Specifically, different studies have used different mathematical formulae to calculate cognitive IIV, which may yield slightly different predictive values and should be considered when interpreting and comparing findings across studies. Instead, cognitive IIV may serve as a sensitive indicator that mental health professionals can use to identify potential cognitive concerns, so that clinical referrals to appropriate specialists can be made. Clarifying this distinction supports the feasibility and relevance of incorporating cognitive IIV into clinical care.

Rodgers’ evolutionary method to concept analysis consists of seven steps: (1) identifying and naming the concept of interest; (2) identifying the surrogate terms and relevant uses of the concept; (3) selecting a sample for data collection (Figure 2); (4) recognizing the attributes of the concept; (5) identifying references, antecedents, and consequence of the concept (Figure 3); and (6) identifying concepts that related to the concepts of interest (Rodgers & Knafl, 2000).

We conducted a literature search from PubMed, Embase, CINAHL Plus with Full Text, and PsycINFO, and using predefined cognitive IIV-related keywords, with inclusion criteria focusing on studies relevant to cognitive IIV in PWH and excluding non-relevant articles. We then applied a concept analysis by extracting key definitions, calculation methods, and findings. Then, we identified themes using an iterative coding process and the recurring concepts were grouped and refined by consensus.

### Literature search

A literature search was conducted on May 28, 2025, using four databases, including PubMed, Embase, CINAHL Plus with Full Text, and PsycINFO. The initial key terms used in these searches for research studies on cognitive IIV in HIV included the terms “cognition,” “intra-individual variability,” “fluctuation,” “dispersion,” “inconsistency,” “HIV,” “people with HIV,” and “human deficiency virus.” Key terms were adapted according to each database’s requirements in consultation with a skilled university librarian (RB), who worked with the research team to ensure that the search parameters accurately reflected the purpose of this concept analysis. The search strategy also included the use of Medical Subject Headings (MeSH) terms where applicable. Detailed search terms for each database are displayed in Table 1. The review included research articles published between 2000 to 2025 to ensure the analysis captured contemporary perspectives on cognitive IIV and avoided reliance on outdated findings. The search yielded 98 articles from PubMed, 286 articles from Embase, 25 articles from CINAHL, and 139 articles from PsycINFO. In total, the initial searches from the databases yielded 548 articles.

The inclusion and exclusion criteria were defined prior to conducting the systematic searches. Studies were included if they met the following criteria: (a) published in English; (b) full-text available; (c) focused on cognitive IIV as a primary variable; (d) PWH identified as the target population; and (e) published after 2000. Studies were excluded if they: (a) did not address cognitive IIV; (b) did not focus on PWH; (c) did not provide full-text access; or (d) were published before 2000.

The preliminary search yielded 548 articles, which were uploaded to Covidence, a software tool for managing literature reviews, for further screening. Duplicates were automatically eliminated (*n* = 131), leaving 417 articles. Two PhD students (PH & HS) independently reviewed these articles in Covidence to ensure consistency in decision making. When discrepancies occurred, a senior reviewer (DEV) acted as an arbitrator to reach a final decision. This preliminary title and abstract review excluded 393 articles that did not provide significant information related to cognitive IIV in the context of PWH, resulting in 24 articles eligible for full-text review. Full-text screening of these 24 articles led to the exclusion of eight additional articles that did not focus on cognitive IIV. Therefore, 16 articles met all inclusion criteria and were included in this concept analysis. Moreover, six additional articles were identified through the references of included articles and gray literature, and were incorporated, resulting in a total of 22 articles included in this concept analysis (see PRISMA diagram in Figure 2). Of the 22 articles included in the current concept analysis, 13 were retained from the prior review by Vance et al. (2022), and nine represent newly published articles identified in the updated search.

## Results

### Surrogate terms and related concepts

Surrogate terms are defined as the exact words or phrases used to describe the concept (Rodgers & Knafl, 2000). Terms commonly used interchangeably with or to describe cognitive IIV in the literature included “cognitive fluctuation,” “dispersion,” “inconsistency,” and “intra-individual variability” (Arce Rentería et al., 2020; Del Bene, Fazeli, et al., 2025; Hines et al., 2016; Vance et al., in press). Figure 3 provides a representation of the attributes, antecedents, and consequences of the concept of cognitive IIV in the context of PWH.

### Inferences of antecedents and consequences

Antecedents are defined as conditions that occur prior to the concept, while consequences are the outcomes that happen afterwards (Rodgers & Knafl, 2000). Some variables function as antecedents in one context while serving as consequences in another. Theoretical frameworks, temporal order, and empirical findings shape these inferences depending on their role within the cause-and-effect process.

Drawing on the larger neuroscience literature, we have interpreted certain cross-sectional findings to suggest that some variables may function as either antecedents or consequences. For example, ART adherence can serve as both an antecedent and a consequence of cognitive IIV in PWH. As an antecedent, Hines et al. (2016) have found that poor adherence to ART may lead to suboptimal viral suppression, contributing to greater cognitive dysfunction, which subsequently contributes to greater cognitive IIV. As a consequence, Mustafa et al. (2023) found that when cognitive IIV is already present, an individual’s ability to remember to take medication or follow treatment plans may be impaired. This bidirectional relationship illustrates how antecedents and consequences are often interwoven and context-dependent, reinforcing the need to examine these roles flexibly when clarifying the concept of cognitive IIV in PWH.

### Antecedents of cognitive IIV in the context of PWH

Eight antecedents of cognitive IIV in the context of PWH were identified: (1) viral load and CD4 count; (2) ART adherence; (3) comorbidities; (4) aging; (5) demographic factors; (6) psychological factors; (7) current substance use; and (8) structural brain changes and brain volume reduction (Figure 3).

### Viral load and CD4 count

Viral load and CD4 count reflect the level of HIV activity and immune function (Anderson et al., 2018; Del Bene, Fazeli, et al., 2025). Higher viral load is associated with greater cognitive IIV, theorized to be due to increased neuroinflammation risk, which can lead to episodic impairments in memory, attention, and executive function (Ettenhofer et al., 2010). Similarly, a declining CD4 count indicates a weakening immune system, making the brain more vulnerable to inflammation-related damage, and associated with worse cognitive performance and greater cognitive IIV (Anderson et al., 2018; Del Bene, Fazeli, et al., 2025). Higher viral loads and lower CD4 counts are believed to result from declines in medication adherence, which can also contribute to greater cognitive IIV (Ettenhofer et al., 2010).

### ART adherence

ART adherence plays a significant role in maintaining cognitive performance (Ettenhofer et al., 2010). Increased cognitive IIV is significantly associated with worse performance on prospective memory tasks, which affect the ability to remember to take medication or attend appointments (Mustafa et al., 2023). Poor and inconsistent ART adherence may result in viral rebound which worsens cognitive outcomes, contributing to greater cognitive IIV (Hines et al., 2016). Additionally, increased cognitive IIV due to inconsistent ART may contribute to challenges in maintaining consistent health behaviors (Morgan et al., 2012; Penheiro, Webber, et al., 2025), which could compromise HIV management and lead to worsened health outcomes (Vance et al., 2022).

### Comorbidities

Comorbidities are commonly found among PWH, and some may contribute to cognitive IIV (Ridgely et al., 2024). Chronic conditions such as cardiovascular disease, metabolic dysregulation (i.e., diabetes), and hypertension were associated with poorer cognitive outcomes as well as greater cognitive IIV in older PWH (Anderson et al., 2018; Hines et al., 2016; Vance et al., in press). The chronic inflammation associated with these comorbidities may further worsen cognitive IIV by accelerating neurodegenerative processes. Additionally, unrelated HIV-comorbidities associated with neuropathological/structural brain changes, such as Parkinson’s disease, Alzheimer’s disease, and subcortical small vessel ischemic disease, may contribute to cognitive performance by increasing cognitive IIV in older PWH (Hines et al., 2016; Vance et al., in press).

### Aging

Older age was a factor influencing both cognitive performance and cognitive IIV in PWH (Levine et al., 2018; Odii et al., 2025; Ridgely et al., 2024; Vance, Azuero et al., 2024). For example, in a study of cognitive IIV among PWH, Levine et al. (2018) found that older PWH exhibited greater cognitive IIV compared to both older PWoH and younger PWH. Prior studies found that older PWH exhibit more variability in cognitive functioning than younger PWH (Morgan et al., 2011; 2012). As people age, the structure of the brain naturally changes, as both gray and white matter volumes decrease in specific cortical regions, including regions involved in cognitive control (i.e., prefrontal cortex). These reductions can lead to less efficient communication within the brain, making it difficult to maintain consistent cognitive performance, resulting in greater cognitive IIV (Clark et al., 2018; Hines et al., 2016; Jones et al., 2018; Vance et al., 2022). As the brain becomes less resilient (the ability to adapt and recover) with age, these age-related changes in brain structure can lead to variability in cognitive performance and may further increase subtle cognitive impairment in older PWH (Del Bene, Fazeli, et al., 2025; Hines et al., 2016).

### Demographic factors

Demographic factors significantly impact cognitive IIV in PWH. In a study of social determinants of health in 131 PWH, Del Bene, Fazeli, et al. (2025) found that lower education levels were associated with greater cognitive IIV (Arce Rentería et al., 2020), suggesting that limited cognitive reserve may predispose individuals to more variability in cognitive performance. Race was another factor that contributed to cognitive IIV. The minoritized racial status contributes to greater cognitive IIV through broader social determinants of health (Arce Rentería et al., 2020). In a study of 131 PWH in the Deep South, Del Bene, Fazeli, et al. (2025) found that greater cognitive IIV was observed in participants who identified as Black or African American, potentially associated with experiences of perceived discrimination. Moreover, participants living below the poverty line demonstrated greater cognitive IIV, suggesting that financial strain may contribute to increased cognitive IIV (Del Bene, Fazeli, et al. (2025). Therefore, increased cognitive IIV due to the negative effects of social determinants of health (e.g., financial strain, discrimination) may result in higher rates of depression, anxiety, and psychological distress, which further impair cognitive functioning and health outcomes (Del Bene, Fazeli, et al., 2025; Penheiro, Kiselica, et al., 2025).

### Psychological factors

Psychological factors significantly influence cognitive IIV in PWH. Mental health issues, such as depression, anxiety, and chronic stress, negatively impact attention, memory, and executive function, leading to greater cognitive IIV (Arce Rentería et al., 2020; Harrison et al., 2017; Vance et al., 2022). In a study of cognitive IIV in PWH, Harrison et al. (2017) found that depression is recognized to independently affect cognition and therefore may increase cognitive IIV. Additionally, higher depression was also associated with reduced cognitive reserve and impaired cognitive functioning (Odii et al., 2025). Such psychological distress may also contribute to ART non-adherence, which may also lead to increased HIV-related neuroinflammation and cognitive impairment. In a study of early life stress and reaction time variability in PWH, Clark et al. (2018) found that increases in cortisol levels due to high exposure to stress may be associated with psychiatric symptoms, contributing to variability in cognitive performance, and leading to increased cognitive IIV.

### Current substance use

PWH with current substance use (e.g., cocaine, methamphetamine) was associated with increased cognitive IIV (Levine et al., 2008; Morgan et al., 2014). PWH who currently use substances demonstrated increased cognitive IIV compared to those without current substance use (Arce Rentería et al., 2020). Arce Rentería et al. (2020) found that HIV infection and substance use disorder both impact the brain’s dopamine system, and when they co-occur, PWH with substance use may worsen dopaminergic dysfunction, potentially driving the greater cognitive IIV as measured across a battery of domain-specific cognitive tests (i.e., dispersion) compared to non-substance users. Additionally, cognitive IIV may be exacerbated by substance use as a comorbid factor (Vance, Azuero et al., 2024). Moreover, tobacco smokers with HIV demonstrated increased cognitive IIV (i.e., inconsistency) in working memory and processing speed tasks (Harrison et al., 2017).

### Structural brain changes and brain volume reduction

As mentioned earlier, greater cognitive IIV has been found to correlated with brain volume reduction. The cortical atrophy (both gray and white matter volume reductions) was associated with greater cognitive IIV (Hines et al., 2016; Vance et al., 2022). Jones et al. (2018) found that reduced integrity of white matter impairs connectivity within frontal-subcortical networks, which leads to inefficient neural communication and increased cognitive IIV. Such reduced volume is an indicator of neuropathology and poorer neurophysiological function, in turn, this is likely what causes contribute to cognitive difficulties in PWH (Clark et al., 2018), which may also be an antecedent and perhaps a consequence of cognitive IIV.

### Attributes of cognitive IIV in the context of PWH

Attributes are defining characteristics of the concept of interest (Rodgers & Knafl, 2000). Three attributes of cognitive IIV in the context of PWH were identified: (1) dysregulation of executive function process; (2) dispersion in cognitive abilities; and (3) inconsistencies in cognitive abilities (Figure 3).

### Dysregulation of executive function process

Dysregulation of executive function refers to impairments in the brain’s ability to manage higher-order cognitive functions (i.e., working memory, goal-directed behavior) (Jones et al., 2018; Ridgely et al., 2024; Vance et al., 2022). Moreover, individuals with poorer performance on tasks of executive function demonstrated greater cognitive IIV, suggesting that cognitive IIV may stem from executive dyscontrol (Ettenhofer et al., 2010; Penheiro, Webber, et al., 2025; Vance et al., in press). In PWH, executive dysregulation is considered a key contributor to cognitive IIV and may lead to difficulties with attention and task management, leading to impairments in everyday functioning (Ridgely et al., 2024).

### Dispersion in cognitive abilities

**Figure 1.**
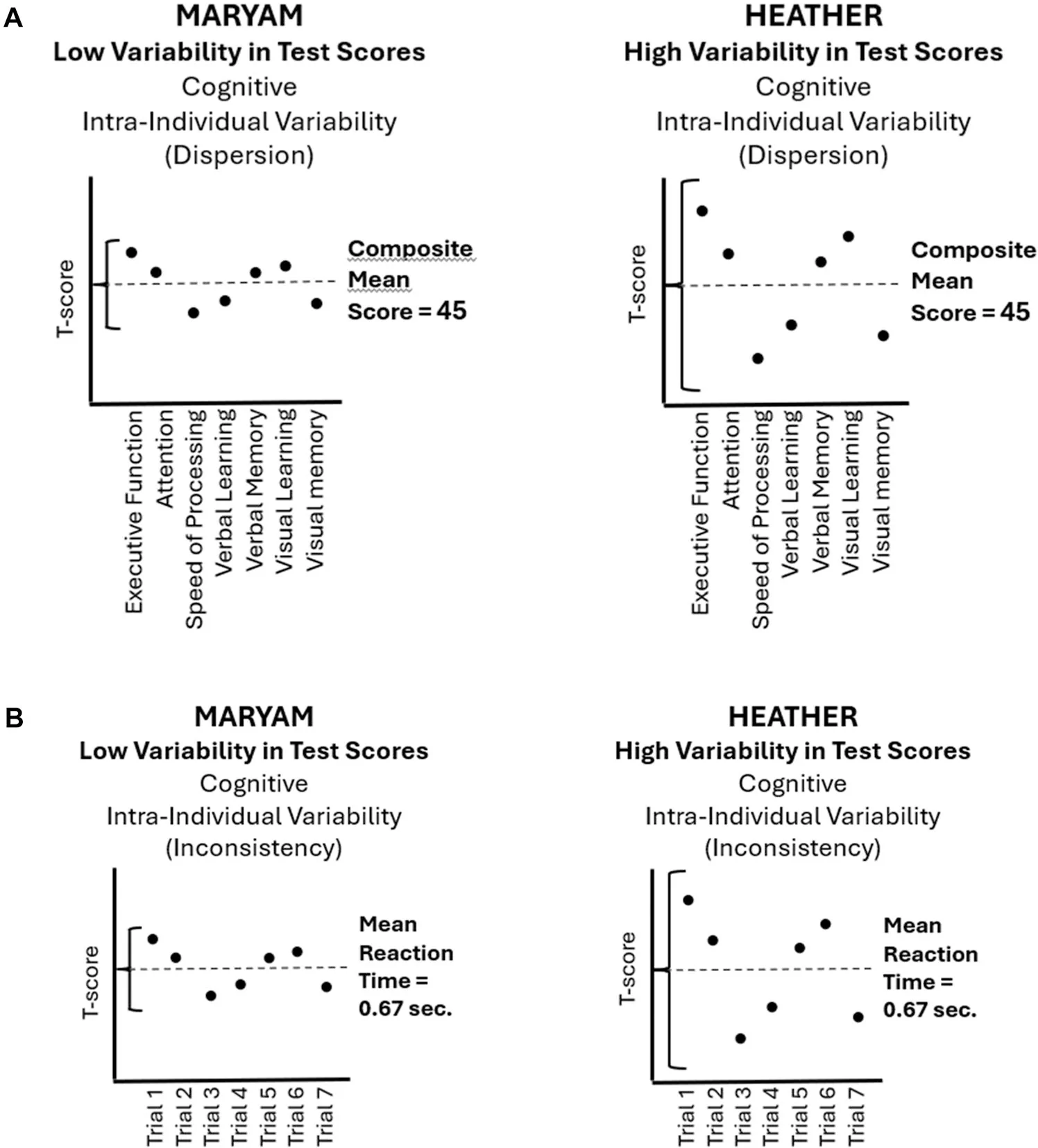
(A) Example of cognitive IIV—dispersion. (B) Example of cognitive IIV—inconsistency.

**Figure 2.**
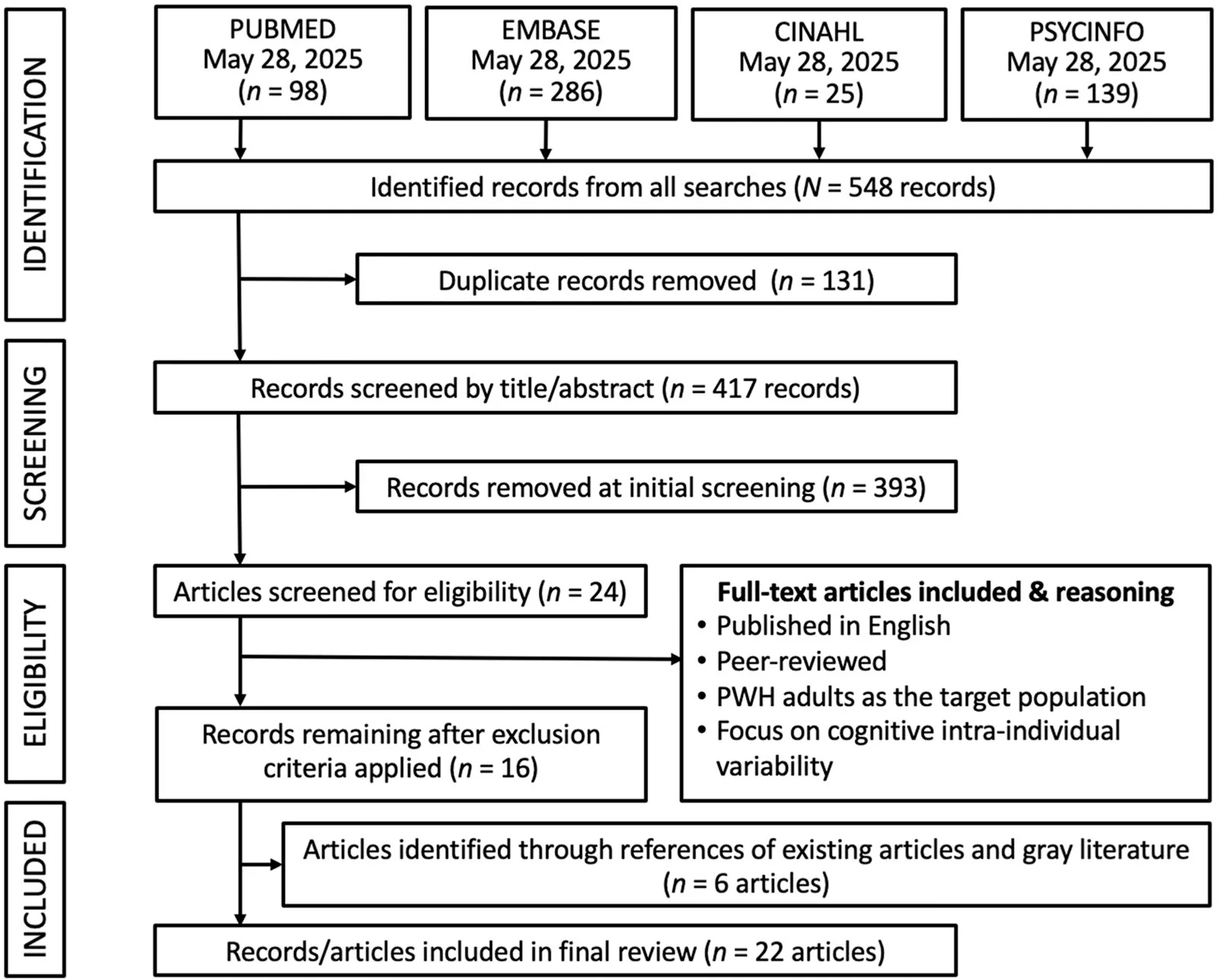
PRISMA flow chart.

**Figure 3.**
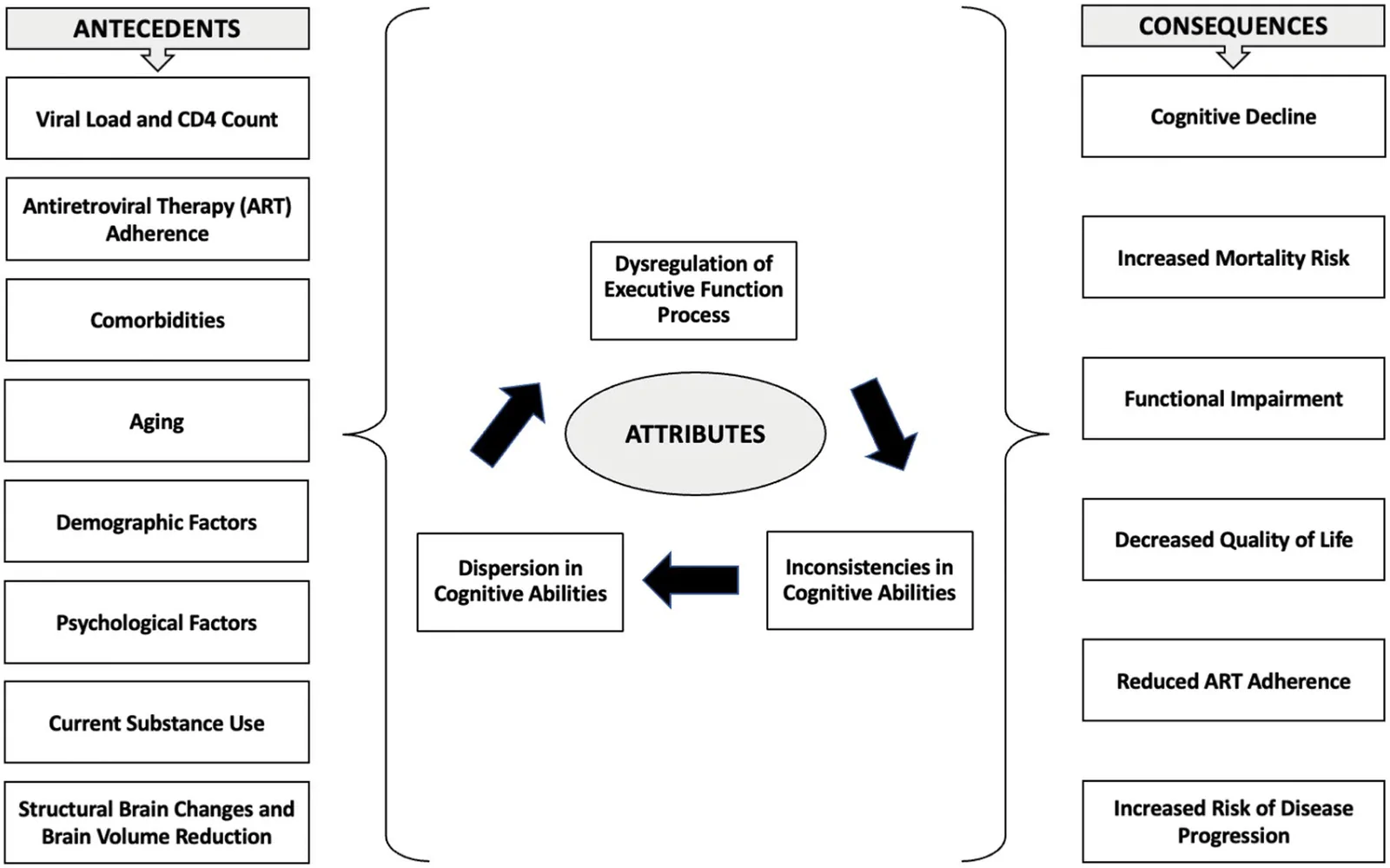
Diagram demonstrating the antecedents, attributes, and consequences of the concept of cognitive IIV in the context of PWH (adapted from Kinnie et al., 2024).

Dispersion (Figure 1A) highlights the variability in an individual’s performance score across cognitive domains (e.g., memory, attention, processing speed, executive function) (Anderson et al., 2018; Penheiro, Webber, et al., 2025; Vance et al., in press). The dispersion scores were calculated as individual standard deviations (iSD) across multiple cognitive domains rather than focusing solely on mean-based performance, and higher dispersion indicates greater cognitive IIV (Del Bene, Fazeli, et al., 2025; Levine et al., 2018; Thaler et al., 2015). Previous studies in PWH have found that elevated dispersion scores have been associated with cortical atrophy in the frontal and temporal lobes and gray matter volume loss, indicating neurobiological compromise (Hines et al., 2016). Higher dispersion has also been associated with poorer cognitive functioning and declines in daily living activities (Morgan et al., 2012; Odii et al., 2025; Vance, Azuero et al., 2024).

### Inconsistencies in cognitive abilities

Inconsistency (Figure 1B) emphasizes variability in an individual’s cognitive performance within the same task (i.e., reaction time variability) (Odii et al., 2025; Thaler et al., 2015; Vance et al., 2022). These inconsistencies are often captured through measures such as reaction time variability, where higher reaction time variability reflects greater inconsistency in cognitive processing speed and attention (Clark et al., 2018). Thus, inconsistency also serves as an indicator of subtle cognitive dysfunction in PWH (Del Bene, Fazeli, et al., 2025).

### Consequences of cognitive IIV in the context of PWH

According to Rodgers and Knafl (2000), consequences are events that result from the existence of a concept. A primary consequence of greater cognitive IIV in PWH is that it likely reflects underlying neurological dysfunction and may serve as an early marker of subsequent cognitive decline and increased risk of dementia (Anderson et al., 2018; Morgan et al., 2011). Over time, greater variability in performance across cognitive domains may signal reduced neural efficiency or stability, which precedes and potentially contributes to progressive decline. For example, in a 14-year longitudinal study of 708 PWH, Anderson et al. (2018) found that participants who presented greater dispersion at one visit or whose dispersion increased substantially between visits developed more severe cognitive impairment at the following assessments. This study also demonstrated that greater cognitive IIV was associated with an increased risk of mortality and served as an early warning sign of death before the next study visit. Additionally, Anderson et al. (2018) suggests that cognitive IIV is conceptualized as a marker of underlying CNS dysfunction rather than an independent causal factor because it is associated with structural brain abnormalities, poor medication adherence, and increased immunosuppression, which may contribute to the clinical pathway leading to mortality.

Another consequence of greater cognitive IIV in PWH was impaired performance in everyday functioning (Arce Rentería et al., 2020; Del Bene, Fazeli, et al., 2025; Harrison et al., 2017; Morgan et al., 2012; Mustafa et al., 2023; Penheiro, Webber, et al., 2025; Ridgely et al., 2024; Vance et al., in press). PWHs with higher cognitive IIV may experience more difficulties in managing daily activities, including missed medication doses and challenges in maintaining independence and engaging in social or occupational roles, resulting in decreased quality of life (Penheiro, Webber, et al., 2025; Ridgely et al., 2024; Vance et al., 2022). Another consequence of greater cognitive IIV in PWH was reduced ART adherence, which could result in increased viral load and lower CD4 counts (Arce Rentería et al., 2020; Ettenhofer et al., 2010; Harrison et al., 2017; Hines et al., 2016; Morgan et al., 2012; Penheiro, Webber, et al., 2025; Ridgely et al., 2024; Thaler et al., 2015; Vance et al., in press). PWH with greater cognitive IIV may experience increased risk of disease progression because of the difficulties in following treatment plans (e.g., missing appointments, managing and remembering to take medication), leading to suboptimal health outcomes (Morgan et al., 2011; 2012; Mustafa et al., 2023; Odii et al., 2025; Vance et al., in press). These disruptions can delay timely interventions and increase the likelihood of worsening health outcomes (Morgan et al., 2012; Penheiro, Webber, et al., 2025; Thaler et al., 2015; Vance et al., 2022).

### Theoretical definition

In HIV care, cognitive IIV is influenced by a complex interaction of biological vulnerability, psychological factors, and cognitive performance, resulting in how reliably the brain consistently functions within a single cognitive domain (i.e., inconsistency) or across cognitive domains (i.e., dispersion). Given how it impacts patient outcomes (e.g., medication adherence), this concept analysis suggested the need to detect cognitive IIV, separately from clinic-based composite cognitive assessments such as the Mini-Mental State Examination (MMSE) or Montreal Cognitive Assessment (MOCA), but from a broad neuropsychological assessment. Furthermore, cognitive interventions that also target cognitive IIV may also be considered to support cognitive stability and improve the overall well-being of PWH.

## Discussion

The current study has extended from an integrative review of cognitive IIV in HIV (Vance et al., 2022) by incorporating nine newly published articles and incorporating a concept analysis approach to further clarify the definitions, attributes, antecedents, consequences, and application of cognitive IIV in PWH. Since the publication of the earlier review, the field has advanced with increased research attention and emerging interventions targeting cognitive IIV, which are integrated into the current concept analysis to provide a more comprehensive and updated understanding of cognitive IIV. Using Rodger’s evolutionary method (Rodgers & Knafl, 2000), eight antecedents, three attributes, and six consequences of cognitive IIV were identified. The antecedents include viral load and CD4 count, ART adherence, comorbidities, aging, demographic factors, psychological factors, current substance use, and structural brain changes and brain volume reduction (Del Bene, Fazeli, et al., 2025; Ettenhofer et al., 2010; Harrison et al., 2017; Vance et al., 2022). For researchers, these results underscore the need to consider cognitive IIV as a dynamic, multifactorial outcome that can reflect a cumulative biopsychosocial burden on brain function.

The attributes of cognitive IIV indicate that executive dysfunction is a key factor contributing to its presence in PWH (Ridgely et al., 2024; Vance et al., 2022). Cognitive IIV may reflect both underlying cognitive dysfunction and state-dependent influences (e.g., motivation, attention). These influences may be distinct but are also potentially interrelated, such as apathy (reduced motivation) which is associated with poorer cognitive and neurological functioning (Totuk & Şahin, 2025). In fact, in HIV, apathy has been associated with grey and white matter pathways and is known to influence cognition and everyday functioning (Thompson et al., 2024). Therefore, cognitive IIV should not be interpreted in isolation. Clinicians should consider contextual factors (e.g., fatigue, mood) and incorporate repeat assessment to determine whether variability reflects transient influences or more persistent neurocognitive dysfunction when appropriate. For clinical practice, recognizing this association may guide cognitive interventions (e.g., executive function training) designed to maintain cognitive performance and improve functional outcomes in PWH.

The consequences of increased cognitive IIV are clinically significant because it impacts daily task management, medication adherence, and overall quality of life. Although various degrees of cognitive IIV are normative, with healthy individuals generally demonstrating relatively coherent performance profiles, there are no fixed cutoffs at which cognitive IIV indicates the presence of cognitive impairment (Del Bene, Aita, et al., 2025). Greater dispersion above the expected range of cognitive IIV may reflect some form of neurological dysfunction. Additionally, standardized reference ranges for normal versus elevated cognitive IIV remain limited, particularly in PWH. Therefore, cognitive IIV should be interpreted as part of normative expectations and the overall clinical presentation. Identifying cognitive IIV as a predictor of functional impairment beyond mean-based cognitive measures provides an opportunity to develop interventions focusing on maintaining and enhancing cognitive performance, such as executive functioning. Understanding cognitive IIV could lead to more proactive and personalized care models, which are essential as PWHs age and face multiple health challenges.

Cognitive IIV is closely related to processing speed, and slow processing speed may contribute to increased variability. However, a recent study suggests that cognitive IIV is not merely a function of processing speed. For example, cognitive IIV did not improve with the processing speed intervention; therefore, it represents a distinct marker of cognition (Vance, Azuero et al., 2024). Additionally, cognitive IIV is associated with neural integrity and mechanisms for controlling attention regardless of processing speed (Burnett et al., 2025). These findings support the interpretation of cognitive IIV as reflecting more general neural efficiency or stability rather than serving solely as a measure of slower processing speed.

Cognitive IIV should be interpreted with consideration of both trait-related and state-related factors. Additionally, cognitive IIV, which is often measured at a single point in time, may not provide an accurate estimate of a person’s usual level of cognitive IIV. Instead, cognitive IIV is likely due to both traits (i.e., the person’s broad range or tendency toward variability) and contextual/state-dependent variation (e.g., fatigue, mood, sleep, caffeine consumption) that can be present at the time of measurement. As a result, estimates from a single assessment will have inherent measurement bias, leading to an overestimation or underestimation of usual variability. This limit applies not just to cognitive IIV, it is one of the inherent limits of most neuropsychological research that commonly uses single-session/cross-sectional methods. Therefore, it is essential to recognize this limitation while interpreting results and emphasize the importance of future studies utilizing repeated or longitudinal assessments to better disentangle stable versus dynamic components of cognitive IIV.

### Clinical implications

The results of this concept analysis underscore the possibility to incorporate cognitive IIV into clinical practice for PWH for early intervention. This context-specific conceptualization is a key contribution, highlighting that cognitive IIV may differ across clinical populations (e.g., PWH, cancer, or aging) and informing its interpretation and clinical application. For example, elevated cognitive IIV is observed across conditions (e.g., PWH, Alzheimer’s disease, cancer), yet these patterns likely arise from distinct underlying mechanisms rather than a single shared process. Similarly, the same type of cognitive profile (e.g., amnestic deficit) can result from different underlying conditions; therefore, increased cognitive IIV across populations should be interpreted within the specific clinical and neurobiological context (Del Bene, Aita, et al., 2025). Further for PWH, even if a patient has normal cognitive functioning but elevated cognitive IIV, this may reflect underlying executive dysfunction related to HIV-associated brain changes that may not have been detected with other executive functioning tests. This interpretation may help identify individuals at risk of medication adherence difficulties and support targeted cognitive interventions. Additionally, there is emerging evidence that interventions targeting executive functioning, such as executive functioning training (EFT), may help stabilize cognitive performance in PWH. In a recent case comparison study of four PWH who either received 20 hours of EFT or no training, those assigned to the EFT experience reduced cognitive inconsistency, indicating more consistent cognitive functioning (Odii et al., 2025). Although these results are preliminary, a study of 119 PWH in this same EFT protocol is currently examining whether EFT can reduce both inconsistency and dispersion; if so, then being able to detect elevated cognitive IIV can be used to indicate possible interventions.

Unfortunately, one of the major limitations of its use in standard care is its feasibility. To enhance feasibility in a clinical setting, brief and practical tools are needed to assess cognitive IIV without requiring extensive neuropsychological batteries. Using cognitive IIV based on reaction time tasks can reduce time constraints and participants’ burden because variability can be measured within a single task rather than across multiple tests, making it more appealing and feasible in clinical settings where contact with patients is brief. But a caveat of that is it would be a mistake to sacrifice specificity of a more comprehensive battery for a quicker measure to get a RT IIV score. Unfortunately, more time-consuming cognitive performance testing is needed to assess cognitive IIV.

### Research implications

The results of this concept analysis underscore the need for future research to investigate cognitive IIV as an early and sensitive marker of cognitive functioning in PWH. As such, a significant implication is that cognitive IIV may help identify individuals most likely to benefit from targeted cognitive interventions, such as cognitive training.

Cognitive training interventions have been explored to enhance cognitive function in PWH who are at risk for cognitive impairment. There are many types of cognitive training interventions and many focus on improving a single cognitive domain, such as executive functioning or memory. For example, in a two-year longitudinal randomized control trial (RCT) called the ThinkFast Study, Vance, Fazeli et al. (2024) assigned 216 PWH 40 and older with HAND or borderline HAND to received: 1) 10 hours of computerized speed of processing (SOP) training; 2) 20 hours of computerized SOP training; or 3) 10 hours of Internet (sham control) training. They found that those who received more SOP training experienced better gains on a SOP measure (i.e., Useful Field of View) which translated to improvements on everyday functioning and quality of life indices. Although this study was not initially designed to look at cognitive IIV, it is hoped that cognitive training interventions could reduce cognitive IIV and perhaps improve cognitive performance.

Interestingly, Vance, Azuero et al. (2024) conducted a secondary data analysis on the ThinkFast Study examining whether cognitive IIV, as measured by dispersion, would function as a moderator or could be changed by the SOP training. They found that cognitive IIV acted as a moderator of the SOP training. In fact, baseline levels of cognitive IIV seemed to influence the short-term and long-term treatment effects of SOP training. Unfortunately, they found that the intervention did not change dispersion. This makes sense when considering that the ThinkFast Study was not initially designed to change cognitive IIV as part of the protocol, but yet, these fascinating findings suggest cognitive IIV should be considered in future cognitive interventions, like the Executive Functioning Training (EFT) study.

In the EFT Study, 119 PWH 40 and older were randomized to either the no-contact control group or to the EFT group where they received 20 hours of executive functioning (Odii et al., 2025). In this RCT, participants received EFT because it was hypothesized that if executive dysfunction could be mitigated, this would improve cognitive IIV. This is the first study (to our knowledge) deliberately designed to reduced cognitive IIV in a clinical population. All participants underwent a baseline and a post-intervention assessment of cognitive IIV in both the dispersion and inconsistency components of cognitive IIV, using standardized cognitive assessment tools. The rationale for this study draws on the Executive Dyscontrol Hypothesis, suggesting that improving executive control may reduce cognitive IIV. Preliminary findings from the EFT Study suggest the following. First, in a case study (*N* = 4) involving two participants assigned EFT and completed all 20 hours of training compared to two demographically matched controls, Odii et al. (2025) found that two cognitive IIV inconsistency indicators (i.e., variability & Hit Reaction Time Standard Deviation (HRTSD)), measured using the Connor’s Continuous Performance Test 3^rd^ Edition (CCPT; 3rd Edition), showed improvement in the training group compared to the no-contact control group, signifying that the training effectively reduced cognitive IIV. Second, in the full sample (*N* = 118), researchers found that PWH in the treatment group showed improvements in variability and HRTSD compared to the control group. The results demonstrated that the executive functioning training seems to be reduced cognitive IIV in cognitively vulnerable PWH (Vance et al., 2025).

Another area for further research is domain-specific inconsistency. During this concept analysis, we recognized that inconsistency is measured by reaction time testing which usually depends on the cognitive domain of attention. In this focus on HIV, inconsistency was not measured in executive, memory, or other cognitive domains. That represents a gap in this area. There are other types of domain-specific cognitive inconsistency in the literature such as motor function (Delmas et al., 2025; Kay et al., 2017) and executive function (Duschek et al., 2022). Currently we do not know the predictive value of different types of different domain-specific inconsistency measures have on clinical outcomes such as cognitive decline, onset of dementia, everyday functioning, and mortality. Further, the procedures in which cognitive tests are structured in other domains, such as verbal fluency and spatial reasoning, do not generate data that lend themselves easily to calculate inconsistency. This may explain why inconsistency measures are not readily observed in other cognitive domains. Thus, this represents an intriguing area for further investigation.

### Strengths and limitations of concept analysis

Clarifying the concept of cognitive IIV through Rodgers’ evolutionary method provided a dynamic approach to identifying the antecedents, attributes, and consequences, resulting in a clearer understanding of its role in HIV care and research. This broadens the ability of mental health professionals to understand and address subtle cognitive dysfunction, provide timely referrals and interventions, and more personalized care to maintain functional independence in PWH. The identification of attributes enhances conceptual clarity and provides a foundation for measurements and interventions.

This concept analysis has two limitations that are endemic to the field of cognitive IIV. First, different approaches were used to define and measure cognitive IIV across studies. This poses challenges in synthesizing findings. As seen in Table 2, although the general conceptual approach was similar, different studies operationalized cognitive IIV differently. While the different methods reflect cognitive IIV, they have slightly different predictive values (Vance et al., in press). To address this limitation, future research should aim to standardize the definition and measurement of cognitive IIV across studies to improve consistency and comparability of findings; or in many cases, studies should include alternative measures and/or calculations of cognitive IIV that will allow cross study comparisons to be made. Second, another inherent limitation in neuropsychology concerns the relationship between mood and cognition. Mood-related factors may impact cognitive performance directly (i.e., neurobiological mechanisms such as HPA axis activation) or indirectly through reduced effort (i.e., motivation, or variable engagement due to internal emotional distraction) during the neuropsychological assessment. Unfortunately, many studies do not account for mood or reported effort in standardized cognitive assessment; thus, the nature of this relationship remains unclear. Currently, unlike accounting for education and age with typical normed based cognitive tests, there is no acceptable way to standardize cognitive IIV, at least for mood and motivation during cognitive assessment. Until such time, we must be cognizant of this intertwined connection and interpret such cognitive information with caution. Although, Kiselica, Kaser et al. (2024) have developed a normative dataset for cognitive IIV for people with Alzheimer’s disease so that clinicians can have some idea of what is considered normal versus abnormal cognitive IIV in a sample of healthy patients. A caveat of this is that the same cognitive battery must be used as the normative data were built from those tests. Perhaps such normative data can be built for other clinical populations such as PWH.

Additionally, a limitation of gathering reliable information on cognitive IIV is that like other cognitive testing, effort involved in cognitive performance may be impacted by the person’s internal state such a mood (i.e., depression), fatigue, or pain, and in some cases cognitive IIV may be elevated due to malingering (Hill & Aita, 2018; Strauss et al., 1999). Thus, embedding assessments of a participant’s or patient’s internal state or performance validity test (PVT) such as the TOMM (Test of Memory Malingering) should be considered to evaluate whether the cognitive performance data are valid and should be used or censured. Thus, using multimodal approaches, such as combining neurocognitive testing with psychological assessments (i.e., PVTs, depression, fatigue, pain) may enhance the rigor and interpretability of results.

## Conclusion

Cognitive IIV provides an innovative approach to understanding moment-to-moment variations in cognitive performance in PWH. It is important to understand that mean-based cognitive testing does not provide the complete picture of one’s cognitive functioning; cognitive IIV provides adjunctive information to consider one’s cognitive health. Moreover, the concept of cognitive IIV holds potential for broader adoption in other clinical groups where executive dysfunction and cognition IIV are shared experiences, such as older adults, and adults with traumatic brain injury and neurodegenerative-related diseases.

## Supplementary Material

Supp 1

Supplemental data for this article can be accessed online at https://doi.org/10.1080/23279095.2026.2694678.

## Figures and Tables

**Table 1.** Terms used to search for targeted articles in PubMed, Embase, CINAHL, and PsycINFO.

Database	Search

PubMed Search conducted on May 28, 2025 Filters: Jan 2000-May 2025 98 results	(“Cognition”[Mesh] OR “Cognitive Dysfunction”[Mesh] OR “Executive Function”[Mesh] OR “Neurocognitive Disorders”[Mesh:NoExp] OR cognit*[tiab] OR neurocogniti*[tiab] OR executive-function*[tiab] OR executive-control*[tiab] OR executive-dysfunction*[tiab]) AND (“Biological Variation, Individual”[Mesh] OR “Individuality”[Mesh] OR dispersion*[tiab] OR fluctuation*[tiab] OR inconsistenc*[tiab] OR IIV[tiab] OR “individual biological variation*”[tiab] OR “individual biological variabilit*”[tiab] OR “intra-individual biological variation*”[tiab] OR “intra-individual biological variabilit*”[tiab] OR “intraindividual biological variation*”[tiab] OR “intraindividual biological variabilit*”[tiab] OR intraindividual-variabilit*[tiab] OR intra-individual- variabilit*[tiab] OR intraindividual-variation*[tiab] OR intra-individual-variation*[tiab] OR “within-person variation*”[tiab] OR “within-subject variation*”[tiab] OR “within-individual variation*”[tiab] OR “within-person variabilit*”[tiab] OR “within-subject variabilit*”[tiab] OR “within-individual variabilit*”[tiab] OR “within-individual biological”[tiab]) AND (“Acquired Immunodeficiency Syndrome”[Mesh] OR “AIDS Dementia Complex/psychology”[MAJR] OR AIDS [tiab] OR “acquired immunodeficiency syndrome”[tiab] OR “HIV”[Mesh] OR “HIV Infections”[Mesh] OR HIV[tiab] OR PLWH[tiab] OR PWH[tiab] OR Human-Immunodeficiency-Virus*[tiab])
Embase Search conducted May 28, 2025 Filters: Jan 2000-May 2025 286 Results	(“cognition”/exp OR “cognitive defect”/exp OR “executive function”/exp OR “disorders of higher cerebral function”/exp OR cognit*:ti,ab OR neurocogniti*:ti,ab OR executive-function*:ti,ab OR executive-control*:ti,ab OR executive-dysfunction*:ti,ab) AND (“biological variation”/de OR “individuality”/de OR “intra individual variability”/exp OR “dispersion”/exp OR dispersion*:ti,ab OR fluctuation*:ti,ab OR inconsistenc*:ti,ab OR IIV:ti,ab OR ((individual-biological OR intra-individual OR intraindividual OR within-person OR within-subject OR individual) NEAR/3 (variation* OR variabilit*)):ti,ab AND (“acquired immune deficiency syndrome”/exp OR “AIDS patient”/exp OR “Human immunodeficiency virus”/exp OR “Human immunodeficiency virus infection”/exp OR “Human immunodeficiency virus infected patient”/exp OR “HIV associated dementia”/exp OR AIDS:ti,ab OR “acquired immunodeficiency syndrome”:ti,ab OR HIV:ti,ab OR PLWH:ti,ab OR PWH:ti,ab OR Human-Immunodeficiency-Virus*:ti,ab)
CINAHL Search conducted on May 28, 2025 Filters: Jan 2000-May 2025 25 results	(MH “Cognition+” OR MH “Cognition Disorders+” OR MH “Executive Function” OR cognit* OR neurocogniti* OR executive-function* OR executive-control* OR executive-dysfunction*) AND (MH “Individuality” OR “intra individual variability” OR “dispersion” OR dispersion* OR fluctuation* OR inconsistenc* OR IIV OR ((individual-biological OR intra-individual OR intraindividual OR within-person OR within-subject OR individual) NEAR/3 (variation* OR variabilit*))) AND (MH “AIDS Dementia Complex” OR MH “AIDS-Related Complex” OR MH “AIDS Patients” OR MH “HIV-Positive Persons+” OR MH “Human Immunodeficiency Virus+” OR MH “HIV Infections+” OR AIDS OR “acquired immune deficiency syndrome” OR HIV OR “Human immunodeficiency virus” OR PLWH OR PWH)
PsycINFO Search conducted on May 28, 2025 Filters: Jan 2000-May 2025; exclude Books 139 results	(MAINSUBJECT.EXACT.EXPLODE(“Cognitive Dissonance”) OR MAINSUBJECT.EXACT.EXPLODE(“Cognitive Impairment”) OR MAINSUBJECT.EXACT.EXPLODE(“Executive Function”) OR cognit* OR neurocogniti* OR executive-function* OR executive-control* OR executive-dysfunction*) AND (MAINSUBJECT.EXACT.EXPLODE(“Individuality”) OR MAINSUBJECT.EXACT.EXPLODE(“Intraindividual Variability”) OR “intra individual variability” OR “dispersion” OR dispersion* OR fluctuation* OR inconsistenc* OR IIV OR ((individual-biological OR intra-individual OR intraindividual OR within-person OR within-subject OR individual) N3 (variation* OR variabilit*))) AND (MAINSUBJECT.EXACT.EXPLODE(“AIDS Dementia Complex”) OR MAINSUBJECT.EXACT.EXPLODE(“AIDS”) OR MAINSUBJECT.EXACT.EXPLODE(“HIV”) OR AIDS OR “acquired immune deficiency syndrome” OR HIV OR “Human immunodeficiency virus” OR PLWH OR PWH)

**Table 2.** Summary of antecedents, attributes, and consequences of cognitive intra-individual variability in HIV care and research articles (*N* = 22).

Study/title	Measure/calculation of cognitive IIV	Antecedents	Attributes	Consequences

1. Anderson et al. (2018) Intraindividual Variability in Neuropsychological Performance Predicts Cognitive Decline and Death in HIV	**Dispersion** - Mathematical formula: Coefficient of Variation (CoV) = within-person standard deviation divided by the within-person mean of 15 cognitive tests' T-scores.	**- Aging:** Older age is a factor influencing both cognitive performance and cognitive IIV. Aging-related neurodegeneration contributes to increased cognitive IIV. **- Comorbidities:** Comorbid conditions (e.g., cardiovascular issues, psychiatric conditions) may influence cognitive IIV and are considered in the context of broader health status. **- Viral Load & CD4 Count:** Higher viral load and lower CD4 count were associated with a greater risk of cognitive dysfunction, which can increase cognitive IIV.	**- Dispersion in Cognitive Abilities:** The dispersion reflects an individual's variability in scores across cognitive domains, such as memory, attention, executive function, etc.	**- Cognitive Decline:** Greater cognitive IIV was significantly associated with a greater likelihood of cognitive decline over time in PWH. Cognitive IIV may predict worsening cognitive function. **- Increased Mortality Risk:** Greater cognitive IIV was associated with an increased risk of death before the next visit. This indicates that cognitive IIV is not only a marker of cognitive IIV but also of broader health vulnerability.
2. Arce Rentería et al. (2020) Neurocognitive Intra-Individual Variability within HIV+ Adults with and without Current Substance Use	**Dispersion** - Mathematical formula: CoV = Intra-individual Standard Deviation (ISD)/ Global T-score (demographically adjusted).	**- Current Substance Use:** PWH with current substance use exhibit greater cognitive IIV compared to those without current substance use. **- Demographic Factors:** **- Education:** Lower education contributes to cognitive IIV through broader social determinants of health. **- Race:** Minoritized racial status contributes to cognitive IIV through broader social determinants of health. **- Psychological Factors:** **- Depression:** Depressive symptoms were considered as potential confounders in the relationship between substance use and cognitive IIV.	**- Dispersion in Cognitive Abilities:** The study interprets increased cognitive IIV as indicating dispersion, particularly among those with current substance use. Elevated dispersion scores were associated with current substance use.	**- Functional Impairment:** Greater cognitive IIV was associated with difficulties in real-world functioning, including problems with attention, decision making, and planning. **- Reduced ART Adherence:** Greater cognitive IIV was associated with poorer medication adherence among PWH with current substance use.
3. Clark et al. (2018) Early Life Stress-Related Elevations in Reaction Time Variability Are Associated with Brain Volume Reductions in HIV+ Adults	**Inconsistency** - Operationalized through RT. - Mathematical formula: CoV=standard deviation (SD) across all correct 1-back trials/mean reaction time (RT).	**- Psychological Factors:** **- Early Life Stress (ELS):** High exposure to ELS was associated with increased cognitive IIV. **- Structural Brain Changes and Brain Volume Reduction:** Elevated RT was associated with reductions of gray and white matter volumes, particularly in the frontal brain region, which may contribute to cognitive difficulties in PWH.	**- Inconsistencies in Cognitive Abilities:** Higher reaction time variability reflects greater inconsistency in cognitive processing speed.	NA
4. Del Bene et al. (2025) Social Determinants of Health and Cross-Sectional Cognitive Intra-Individual Variability in Adults from the Deep South Living with HIV	**Dispersion** - Mathematical formula: CoV = ISD/OTMB (overall test battery mean) from demographically adjusted T-scores).	**- Demographic Factors:** **- Education:** Lower education was associated with greater cognitive IIV, suggesting limited cognitive reserve predisposes individuals to more variability in performance. **- Income:** participants living below the poverty line had greater cognitive IIV, highlighting economic disadvantage (financial strain) as a precursor to cognitive variability. **- Race:** Greater cognitive IIV was found in participants who were Black/African American (perceived discrimination). **- Viral Load & CD4 Count:** Higher viral load and lower CD4 count were associated with poorer cognition and greater cognitive IIV.	**- Dispersion in Cognitive Abilities:** The study measured cognitive IIV across seven cognitive domains, highlighting that variability can span across multiple cognitive domains.	**- Functional Impairment:** Greater cognitive IIV was associated with higher risk of functional impairments, especially in complex tasks (e.g., medication adherence, self-management).
5. Ettenhofer et al. (2010) Reaction Time Variability in HIV-Positive Individuals	**Inconsistency** - Measured by using multiple RT variability metrics from the Conners' Continuous Performance Test-II (CPT-II).	**- ART Adherence:** Elevated RT variability was associated with poorer ART adherence. **- Viral Load & CD4 Count:** Higher viral load and lower CD4 count were associated with greater cognitive IIV due to increased neurological risk.	**- Dysregulation of Executive Function Process:** Increased RT variability was associated with poorer performance on tasks of executive function, suggesting that cognitive IIV may stem from executive dyscontrol. **- Inconsistencies in Cognitive Abilities:** The inconsistency reflects moment-to-moment variability in cognitive domains (e.g., attention, processing speed).	**Functional Impairment:** Increased RT variability in PWH was associated with subtle cognitive impairment that negatively impacts daily activities. **- Reduced ART Adherence:** Increased RT variability contributed to the declines in ART adherence.
6. Harrison et al. (2017) The Nature and Consequences of Cognitive Deficits among Tobacco Smokers with HIV: A Comparison to Tobacco Smokers without HIV	**Inconsistency** - Mathematical formula: CoV = SD of RT on the CPT and n-back tests divided by the mean RT; this reflects IIV in RT.	**- Current Substance Use:** PWH who smoke demonstrated inconsistency on measures of working memory and processing speed. **- Psychological Factors:** **- Depression:** Higher depression ratings in PWH smokers were associated with lower working memory performance. **- Viral Load & CD4 Count:** A higher CD4 count was associated with lower working memory, suggesting that PWH with poorer working memory may exhibit greater variability in their cognitive performance.	NA	**- Functional Impairment:** Increased cognitive IIV was associated with poorer performance on daily functioning. **- Reduced ART Adherence:** Cognitive deficits in PWH, especially those who smoke, were associated with poor ART adherence.
7. Hines et al. (2016) Cortical Brain Atrophy and Intra-Individual Variability in Neuropsychological Test Performance in HIV Disease	**Dispersion** - Measured by calculating ISD from the standardized performance scores of five cognitive tests.	**- Aging:** Older age was associated with increased cognitive IIV, suggesting that age-related changes in brain structure may influence variability in cognitive performance. **- Comorbidities:** Cardiovascular disease, metabolic dysregulation, Alzheimer's disease, and subcortical small vessel ischemic disease were associated with variability in cognitive performance in older PWH. **- Structural Brain Changes and Brain Volume Reduction:** Greater cognitive IIV was associated with atrophy of gray matter volume in specific cortical regions (e.g., inferior frontal gyrus).	**- Dispersion in Cognitive Abilities:** Dispersion was positively associated with cortical atrophy, particularly in frontal and temporal regions, and correlated with gray matter volume loss in specific cortical regions.	**- Functional Impairment:** Greater cognitive IIV was associated with poorer performance in activities of daily living. **- Reduced ART Adherence:** Greater cognitive IIV was associated with poor and inconsistent ART adherence.
8. Jones et al. (2018) Longitudinal Intra-individual Variability in Neuropsychological Performance Relates to White Matter Changes in HIV	**Dispersion** - Measured by calculating ISD of the 14 neuropsychological test scores from each participant.	**- Aging:** Age-related decline in white matter integrity is associated with less efficient neural connectivity and increased variability in cognitive performance over time. **- Structural Brain Changes and Brain Volume Reduction:** Reduced integrity of white matter impairs connectivity within frontal-subcortical networks, leading to inefficient neural communication and increased cognitive IIV.	**- Dispersion in Cognitive Abilities:** The study operationalized cognitive IIV as the standard deviation of 14 cognitive test scores, reflecting variability across cognitive domain at a single time point. **- Dysregulation of Executive Function Process:** Increased cognitive IIV reflects frontal-subcortical dysfunction, where lapses in attention and self-monitoring lead to variable performance across cognitive measures.	**- Cognitive Decline:** Greater cognitive IIV was significantly associated with reduced white matter integrity, demonstrated greater sensitivity to neurologic changes in PWH.
9. Levine et al. (2008) Elements of Attention in HIV infected Adults: Evaluation of an Existing Model	**Inconsistency** - Measured by variability in RT and omission errors on the CPT.	**- Current Substance Use:** PWH with current substance use (e.g., cocaine, methamphetamine) is associated with greater cognitive IIV.	NA	**- Cognitive Decline:** Greater cognitive IIV was associated with poorer sustained attention and variability in cognitive performance over time. **- Functional Impairment:** Greater cognitive IIV was associated with difficulties in real-world tasks requiring sustained attention and stable performance. **- Reduced ART Adherence:** Greater cognitive IIV was associated with poorer adherence to ART.
10. Levine et al. (2018) Intraindividual Variability in Neurocognitive Performance: No Influence Due to HIV Status or Self-Reported Effort	**Dispersion** - Measured by using the calculated ISD of an individual's test scores across multiple domains (i.e., working memory, etc.).	**- Aging:** Older age was associated with greater cognitive IIV.	**- Dispersion in Cognitive Abilities:** Dispersion was a reliable indicator of cognitive dysfunction that is not due to suboptimal effort; however, it is not specific to HIV.	NA
11. Morgan et al. (2011) Intraindividual Variability in HIV Infection: Evidence for Greater Neurocognitive Dispersion in Older HIV Seropositive Adults	**Dispersion** - Measured by calculating ISD across Z-scores from 12 measures.	**- Aging:** Older PWH demonstrated greater variability in cognitive performance than younger PWH and both older and younger without HIV infection. **- Viral Load & CD4 Count:** Higher viral load and lower CD4 count were associated with greater cognitive IIV.	**- Dispersion in Cognitive Abilities:** Greater dispersion reflected cognitive dyscontrol in PWH, which was driven by the combined effects of aging and HIV infection on prefrontostriatal systems. Dispersion can be used as a sensitive early indicator of cognitive changes that may not yet be detected by standard screening measures.	**- Cognitive Decline:** Greater dispersion was associated with cognitive decline over time in PWH. **- Functional Impairment:** Greater cognitive IIV was associated with poorer performance on everyday functioning, such as tasks requiring sustained attention and planning. **- Reduced ART Adherence:** Greater cognitive IIV was associated with managing medications, which could affect ART adherence.
12. Morgan et al. (2012) Intra-individual Neurocognitive Variability Confers Risk of Dependence in Activities of Daily Living among HIV-Seropositive Individuals without HIV-Associated Neurocognitive Disorders	**Dispersion** - Measured by calculating ISD across 13 cognitive test scores.	**- Aging:** Older PWH exhibit more variability in cognitive functioning.	**- Dispersion in Cognitive Abilities:** Dispersion provides additional information beyond average cognitive performance, capturing variability that may impact cognitive abilities. Elevated dispersion scores were associated with declines in activities of daily living, highlighting the sensitivity of cognitive IIV to functional outcomes.	**- Functional Impairment:** Greater cognitive IIV was associated with greater dependence in instrumental activities of daily living (IADLs) (e.g., medication management, financial planning, social responsibilities), suggesting that cognitive IIV reflects early functional impairment. **- Reduced ART Adherence:** Greater cognitive IIV was associated with declines in ART adherence among PWH, suggesting that greater cognitive IIV may contribute to challenges in maintaining consistent health behaviors.
13. Morgan et al. (2014) Elevated Intraindividual Variability in Methamphetamine Dependence Is Associated with Poorer Everyday Functioning	**Inconsistency** - Measured by variability in RT across the Conner's CPT.	**- Current Substance Use:** PWH with current substance use (e.g., cocaine, methamphetamine) is associated with increased cognitive IIV.	NA	**- Functional Impairment:** Greater cognitive IIV was associated with poorer performance on sustained attention tasks, which are important for everyday functioning. **- Reduced ART Adherence:** Greater cognitive IIV was associated with poorer adherence to ART.
14. Mustafa et al. (2023) Lower Prospective Memory is Associated with Higher Neurocognitive Dispersion in Two Samples of People with HIV: A Conceptual Replication Study	**Dispersion** - Mathematical formula: CoV = ISD ÷ IM (intra-individual mean) of T-scores across multiple cognitive tests.	**- Aging:** Older age was considered as a potential factor influencing cognitive IIV. **- Comorbidities:** Various medical comorbidities (e.g., hepatitis C, cardiovascular disease) can impact brain structure and function, leading to prospective memory deficits and increased dispersion. **- Demographic Factors:** **- Education:** Education levels were considered as a potential factor influencing cognitive IIV. **- Prospective Memory Deficits:** Lower scores on prospective memory performance were significantly associated with greater dispersion.	**- Dispersion in Cognitive Abilities:** Greater dispersion was associated with lower scores on prospective memory tasks, suggesting that prospective memory performance may be an early marker of cognitive IIV.	**- Functional Impairment:** Greater cognitive IIV was associated with poorer prospective memory, which reflects challenges in everyday functioning, such as remembering to perform future intended actions. **- Reduced ART Adherence:** Greater cognitive IIV was significantly associated with worse performance on prospective memory tasks, which affect the ability to remember to take medication or attend appointments.
15. Odii et al. (2025) Executive Functioning Training for Reducing Cognitive Intra-Individual Variability in People Living with HIV: A Pilot Randomized, Controlled Trial Protocol	**Dispersion** - Generated by measuring fluctuation in cognitive variability across cognitive domains using the BRACE+. **Inconsistency** - Measured by variability in RT using the Conner's CPT.	**- Aging:** Older age was associated with worsening cognitive function and increased cognitive IIV.	**- Dispersion in Cognitive Abilities:** Greater dispersion was reported to be associated with poorer cognitive functioning, suggesting that executive dysfunction may contribute to increased cognitive IIV. **- Inconsistencies in Cognitive Abilities:** The inconsistency reflects within-task variability.	NA
16. Odii et al. (2025) A Brief Report of an Executive Functioning Training Pilot RCT in Adults with HIV: A Descriptive Case Comparison Study	**- Dispersion** - Defined as variability across different cognitive tests; not measured in this study. **Inconsistency** - Measured by variability in RT using the Conner's CPT.	**- Aging:** Older age was associated with cognitive decline and increased cognitive IIV. **- Psychological Factors:** **- Depression:** Higer depression was associated with reduced cognitive reserve and impaired cognitive functioning.	**- Inconsistencies in Cognitive Abilities:** The inconsistency reflects within-task variability across trials.	**- Cognitive Decline:** Greater cognitive IIV was associated with poorer cognitive performance.
17. Penheiro et al. (2025) Executive Functions Are Independently Associated with Cognitive Dispersion in HIV Disease	**Dispersion** - Mathematical formula: CoV = intra-individual standard deviation (ISD) of T-scores/mean T-score across the six Cogstate subtests.	NA	**- Dispersion in Cognitive Abilities:** Greater dispersion was associated with poorer executive functioning, suggesting that disrupted executive control processes are a key contributor to cognitive IIV.	**- Functional Impairment:** Greater cognitive IIV was associated with poorer everyday functioning, suggesting that greater cognitive IIV in PWH may contribute to challenges in decision making and independent living. **- Reduced ART Adherence:** Increased cognitive IIV was associated with declines in ART adherence, suggesting that greater cognitive IIV may contribute to challenges in maintaining consistent health behaviors.
18. Ridgely et al. (2024) Cognitive Intra-individual Variability in the Laboratory is Associated with Greater Executive Dysfunction in the Daily lives of older Adults with HIV	**Dispersion** - Mathematical formula: coV=dividing each participanťs IsD by their mean.	**- Aging:** Older age was associated with cognitive decline and considered as a potential factor that can contribute to increased cognitive IIV.	**- Dispersion in Cognitive Abilities:** Greater dispersion was associated with different aspects of executive dysfunction, including the subdomains of planning, decision making, cognitive flexibility, etc.	**- Decreased Quality of Life:** Greater cognitive IIV was associated with a decrease in everyday functioning, resulting in poorer quality of life. **- Functional Impairment:** Greater cognitive IIV was associated with increasing risk of activities of daily living (ADLs) dependence.
19. Thaler et al. (2015) Increased Neurocognitive Intra-individual Variability is Associated with Declines in Medication Adherence in HIV-Infected Adults	**Dispersion** - Measured by 1) calculated the standard deviation of T-scores across 18 cognitive tests at both baseline and 6-month follow-up. 2) a change score (DispersionΔ) was computed to assess how dispersion changed over time: DispersionΔ = (6-month dispersion - baseline dispersion) / baseline dispersion.	**- Aging:** Aging was a potential factor influencing both cognitive variability and medication adherence in PWH. **- Psychological Factors:** **- Depression:** Higher levels of depressive symptoms were associated with increased cognitive IIV and predicted poorer medication adherence.	**- Dispersion in Cognitive Abilities:** Higher dispersion scores indicated greater variability, and dispersion may serve as an indirect indicator of cognitive changes in pWH.	**- Functional Impairment:** Greater cognitive IIV was associated with poorer daily functioning and health behaviors, such as poor adherence to ArT. **- Increased Risk of Disease Progression:** Greater cognitive IIV was associated with increased risk of disease progression and treatment failure, potentially due to poor ArT adherence. **- Reduced ART Adherence:** Greater cognitive IIV was associated with poor ART adherence in PWH.
20. Vance et al. (2022) cognitive intra-individual Variability in HIV: An integrative review	**Dispersion** - Eight studies used a cognitive battery with various tests to calculate dispersion. - Various calculations were used. **Inconsistency** - Five studies used either the CPT (n = 3) OR the n-back test (n = 2) to calculate inconsistency. - Various calculations were used.	**- Aging:** Older age was associated with increased cognitive IIV. **- Demographic Factors:** **- Education:** Lower education levels were associated with greater cognitive IIV. **- Structural Brain Changes and Brain Volume Reduction:** Greater cognitive IIV was associated with cortical atrophy (both gray and white matter volume reductions).	**- Dispersion in Cognitive Abilities:** Higher dispersion was associated with poorer cognitive functioning. **- Inconsistencies in Cognitive Abilities:** Higher inconsistency was associated with poorer cognitive functioning.	**- Cognitive Decline:** Greater cognitive IIV was associated with cognitive decline and may predict worsening cognitive function. **- Decreased Quality of Life:** PWH with greater cognitive IIV may experience more difficulties in maintaining independence and engaging in social OR occupational roles, affecting overall quality of life. **- Functional Impairment:** Greater cognitive IIV was associated with poorer everyday functioning. **- Increased Mortality Risk:** Greater cognitive IIV was associated with an increased risk of mortality. **- Reduced ART Adherence:** Greater cognitive IIV was associated with poor ArT adherence.
21. Vance et al. (2024) Cognitive Intra-Individual Variability as an outcome OR Moderator of speed of processing Training in Aging Adults with HiV-Associated Neurocognitive Disorder: A secondary data analysis of a 2-Year Longitudinal Randomized Clinical Trial	**Dispersion** - Mathematical formula: CoV = ISD ÷ IM (intra-individual mean) of T-scores across seven cognitive domains.	**- Aging:** Older age was associated with cognitive decline and increased cognitive IIV. **- Current Substance Use:** Cognitive IIV may be exacerbated by substance use as a comorbid factor.	**- Dispersion in Cognitive Abilities:** Greater cognitive IIV (dispersion) was associated with poorer cognitive functioning.	**- Cognitive Decline:** Greater cognitive IIV was associated with poorer cognitive functioning and progression of cognitive impairment as measured by a visual speed of processing test.
22. Vance et al. (in press) Does Cognitive Intra-Individual Variability predict change in Everyday Functioning performance in Women with and without HIV in the Women's Interagency HIV Study?	**Dispersion** - Measured by using two different calculations: 1) ISD); and 2) coV. - Mathematical formulas: 1) ISD is defined as the standard deviation of *Z-score* for the seven cognitive domains. 2) CoV is defined as the standard deviation divided by the mean for each of the seven cognitive domains.	**- Aging:** older age contributed to greater cognitive IIV.	**- Dispersion in Cognitive Abilities:** Higher dispersion was indicated poor coordination of cognitive abilities, potentially signaling subtle cognitive decline.	**- Functional Impairment:** Increased cognitive IIV could place individuals at greater risk for loss of independence, resulting in difficulties in everyday functioning. Higher cognitive IIV could predict greater decline in everyday functioning, including difficulties in performing IADLs (e.g., managing finances, medication, transportation). **- Reduced ART Adherence:** Greater cognitive IIV was associated with difficulties in managing medication, leading to poorer ART adherence.

*Notes.* ADL = activities of daily living; ART = antiretroviral therapy; BRACE+ = Brainbaseline Assessment of Cognition and Everyday functioning; CoV = coefficient of variation; CPT = conner's continuous performance test; ELS = early life stress; IADL = instrumental activities of daily living; IIV = intra-individual variability; IM = intra-individual mean; ISD = intra-individual standard deviation; PWH = people with HIV; OTBM = overall test battery mean; RT = reaction time; SD = standard deviation.

## Data Availability

This is a literature review. We have provided the search parameters and mesh terms (Table 1) whereby others can replicate the search and reproduce the findings. Actual tabulation of the reviewed articles and summaries of the articles are provided in this systematic review in Table 2.
