# Supplementary material for: Exploring the potential of cognitive intra-individual variability in HIV care and research in adults with HIV: a concept analysis
Source: Appl Neuropsychol Adult. Author manuscript; Available in PMC 2026 Aug 25. (PMC13504738; doi:10.1080/23279095.2026.2694678)
Supplement: Supplementary file 1 [file NIHMS2197358-supplement-Supp_1.docx]

**Declaration of Interest Statement**

The authors report no real or perceived vested interests related to this article that could be construed as a conflict of interest.
